# Clinical features and effectiveness of Chinese medicine in patients with COVID-19 from overseas: A retrospective study in Xiamen, China

**DOI:** 10.3389/fpubh.2022.1038017

**Published:** 2022-10-24

**Authors:** Yu-Xuan Huang, Na-Fen Li, Chen-Yao Li, Fang-Ping Zheng, Xiang-Yang Yao, Bao-Hua Lin, Xian-Zhong Huang, Neng-Jiang Zhao, Jia-Yong Yang, Qiu-Min Chen, Man-Man Zhang, Li-Tao Yi, Xue-Qin Chen

**Affiliations:** ^1^The First Affiliated Hospital of Xiamen University, Xiamen, China; ^2^Fujian University of Traditional Chinese Medicine, Fuzhou, China; ^3^Department of Traditional Chinese Medicine, Xiamen Maluan Wan Hospital, Xiamen, China; ^4^Xinglin Branch of the First Hospital of Xiamen University, Designated Hospital for Treatment of Novel Coronavirus Pneumonia in Xiamen, Xiamen, China; ^5^Department of Chemical and Pharmaceutical Engineering, Huaqiao University, Xiamen, China

**Keywords:** COVID-19, SARS-CoV-2, Chinese medicine, retrospective study, network pharmacology, bioinformatics

## Abstract

COVID-19, referred to as new coronary pneumonia, is an acute infectious disease caused by a new type of coronavirus SARS-CoV-2. To evaluate the effect of integrated Chinese medicine and Western medicine in patients with COVID-19 from overseas. Data were collected from 178 COVID-19 patients overseas at First Affiliated Hospital of Xiamen University from April 1, 2021 to July 31, 2021. These patients received therapy of integrated Chinese medicine and western medicine. Demographic data and clinical characteristics were extracted and analyzed. In addition, the prescription which induced less length of PCR positive days and hospitalization days than the median value was obtained. The top 4 frequently used Chinese medicine and virus-related genes were analyzed by network pharmacology and bioinformatics analysis. According to the chest computed tomography (CT) measurement, abnormal lung findings were observed in 145 subjects. The median length of positive PCR/hospitalization days was 7/7 days for asymptomatic subjects, 14/24 days for mild subjects, 10/15 days for moderate subjects, and 14/20 days for severe subjects. The most frequently used Chinese medicine were *Scutellaria baicalensis* (Huangqin), *Glycyrrhiza uralensis* (Gancao)*, Bupleurum chinense* (Chaihu), and *Pinellia ternata* (Banxia). The putative active ingredients were baicalin, stigmasterol, sigmoidin-B, cubebin, and troxerutin. ACE, SARS-CoV-2 3CL, SARS-CoV-2 Spike, SARS-CoV-2 ORF7a, and caspase-6 showed good binding properties to active ingredients. In conclusion, the clinical results showed that integrated Chinese medicine and Western medicine are effective in treating COVID-19 patients from overseas. Based on the clinical outcomes, the putative ingredients from Chinese medicine and the potential targets of SARS-CoV-2 were provided, which could provide a reference for the clinical application of Chinese medicine in treating COVID-19 worldwide.

## Introduction

COVID-19, referred to as new coronary pneumonia, is an acute infectious disease caused by a new type of coronavirus (SARS-CoV-2) at the end of 2019. It has the characteristics of strong transmission and rapid infection. Generally, people suffering from COVID-19 have a wide range of symptoms, including cough, diarrhea, sore throat, and fever (1). On March 11, 2020, the World Health Organization announced that COVID-19 is a global human-scale acute infectious disease epidemic (2). Globally, there have been more than 599 billion confirmed cases of COVID-19, including more than 6.4 million deaths, according to the report by WHO. Currently, more than 10 vaccines have been approved for marketing or emergency use worldwide, and a total of 12.45 billion people have been vaccinated worldwide. However, the global COVID-19 epidemic is still severe and has not been fully controlled. Besides, the frequent occurrence of SARS-CoV-2 mutation brings new challenges (3, 4).

Globally, WHO has developed several clinical practice guidelines for therapeutics for people suffering from COVID-19 (5). In addition to the active chemical ingredient, WHO also evaluated the contribution of traditional medicine integrated approach in the treatment of COVID-19 and approved a protocol for testing herbal medicines as potential treatments for COVID-19 (6, 7). In China, since the emergence of COVID-19, the government has implemented a series of preventive control and medical treatment measures in time, including a cooperative treatment strategy composed of Western medicine and Chinese medicine (8). Chinese medicine actively participates in global epidemic prevention and treatment (9). The unique advantages and characteristics of the “Chinese plan” have been brought into play in all stages of prevention, treatment, and rehabilitation (10). In the stage of epidemic prevention and control, Chinese medicine experts participated in the whole process in the early stage, starting from the study of the characteristics of the virus strain, summing up experience, revising the Chinese medicine epidemic prevention plan, and closely cooperating with Chinese medicine and Western medicine (11). The implementation of classified and precise treatment has been verified to reduce the morbidity rate, reduce the time of negative conversion, reduce hospitalization time, and reduce the rate of conversion to severe disease (12). In real-world research, Lianhua Qingwen capsules are widely used for COVID-19. Several randomized controlled trials have found Lianhua Qingwen capsules have clinical advantages in the treatment of COVID-19, providing effective symptomatic relief to improve the prognosis of life (13, 14). In addition, it was found that Qingfei Paidu decoction combined with conventional treatment also attenuated clinical symptoms in COVID-19, and no severe adverse reactions associated with Qingfei Paidu decoction were observed (15).

As the main entry port for overseas fixed flights, Xiamen has undertaken the treatment of a large number of overseas COVID-19 patients. In the present study, a total of 223 overseas COVID-19 patients were collected in Xiamen from April 1, 2021, to July 31, 2021. This retrospective study, for the first time, analyzes and summarizes the use of integrated Chinese medicine and Western medicine in COVID-19 patients from overseas, which could provide a reference for the clinical application of Chinese medicine in the treatment of COVID-19 worldwide.

## Patients and methods

### Study design

This retrospective study was conducted at the First Affiliated Hospital of Xiamen University in Jimei District of Xiamen city. After a careful chart review, we collected all the patients diagnosed with COVID-19 between April 1, 2021, and July 31, 2021. The cases which did not have complete examination were excluded. The baseline date was set as the date of hospital admission. Patients were diagnosed with COVID-19 using RT-PCR assays by testing the samples from nasopharyngeal swabs. Positive SARS-CoV-2 infective patients received integrated Chinese and Western medicine. All studies strictly followed the Chinese COVID-19 guidelines for diagnosis, admission, and discharge. The study was approved by the ethics review committee of the First Affiliated Hospital of Xiamen University (No. 2021-045) and carried out in accordance with the Helsinki Declaration of the World Medical Association. The written informed consent of patients was waived in this retrospective study.

### Disease severity

Clinicians defined disease severity according to the latest Chinese COVID-19 diagnosis and treatment guidelines. Briefly, asymptomatic was defined as the absence of symptoms but the presence of SARS-CoV-2 infection by RT-PCR assay. Mild disease was defined as the presence of mild symptoms (cough, sore throat, fever, expectoration, runny nose, myalgias, etc.) without viral pneumonia or hypoxia. Moderate disease was defined as the presence of non-severe pneumonia symptoms (cough, fever, dyspnea, shortness of breath, and invagination of the chest). Besides, moderate disease was distinguished from mild disease assisted by computed tomography (CT) of the chest. Severe was defined as symptoms of respiratory rate more than 30 times/min, blood oxygen saturation <93%, the partial pressure of oxygen in arterial blood <300 mmHg, or pulmonary CT showing that the lesions progressed significantly within 24–48 h.

### Treatment

Patients with COVID-19 in the integrated Chinese medicine and Western medicine group received a personalized Chinese decoction (400 mL 2 times daily) and fixed combination of Arbidol/α-interferon (600 mg and 5 million U). The composition, compatibility, and dosage were adjusted according to the progression of clinical symptoms and individual physical differences. Each formula yielded 800 mL of decoction. The trained technicians prepared the decoction during the therapeutical period according to standardized procedures.

### Baseline data and the primary endpoint

Demographic baseline data of positive SARS-CoV-2 infective patients, including age, sex, and basic disease, were collected after a careful inquiry and review. Then, the patients were evaluated by measuring chest CT, number of leukocytes, number of lymphocytes, and C-reactive protein (CRP) levels. The primary endpoint of the present study was viral clearance. After initiation of treatment, SARS-CoV-2 was tested by RT-PCR on nasopharyngeal swabs daily. After getting a negative RT-PCR report, another RT-PCR test was used for confirmation the other day.

### Identification of active compounds and target genes of Chinese medicine in the treatment of COVID-19

The top four frequently used Chinese medicine were chosen for bioinformatics research. Relevant chemical ingredients were collected from the TCMSP website. Candidate ingredients were screened according to the conditions of oral bioavailability ≥ 30% and drug-likeness ≥ 0.18. PharmMapper, a large pharmacophore database, was used to predict the human protein target genes. Target genes with Fit-Score ≥ 0.8 are retained as potential drug targets. GeneCards and DisGeNet database were searched with “Novel Coronavirus Pneumonia” “viral pneumonia” “COVID-19” “2019-nCoV” to obtain the COVID-19 related genes. By drawing a Venn diagram, the intersection of the COVID-19-related genes and Chinese medicine targets was selected as anti-COVID-19 genes.

### Construction of protein-protein interaction (PPI) and drugs-ingredients-targets network

Multiple proteins section of STRING was used to visualize the Homo sapiens genes relationship among anti-COVID-19 genes. The PPI network was imported into Cytoscape 3.8.2 software, and the online topological analysis of the network was performed using the “Network Analysis” function in Tools to calculate the core genes. At the same time, it could classify multiple groups of drugs, ingredients, and targets and get a “drug-ingredient-target” network by setting different colors and shapes.

### Gene ontology (GO) and Kyoto encyclopedia of genes and genomes (KEGG) analysis

The Metascape database was used to discover the related process of anti-COVID-19 genes of TCM in treating COVID-19. The Gene Symbol section was chosen for a personalized analysis. GO enrichment divides into GO Molecular Functions (MF), GO Biological Processes (BP), and GO Cellular Components (CC). The pathway of anti-COVID-19 genes in the treatment of COVID-19 was enriched by KEGG. The threshold of *P*-value Cutoff = 0.01 and Min Enrichment = 1.5 was set to discover the major signaling pathways.

### Molecular docking

The 3D structures of SARS-CoV-2-related proteins (ACE, ACE2, SARS-CoV-2 3CL, SARS-CoV-2 helicase-NSP13, SARS-CoV-2 Spike, SARS-CoV-2 NSP9, SARS-CoV-2 Nucleocapsid Phosphoprotein, SARS-CoV-2 ORF3a, SARS-CoV-2 ORF7a, SARS-CoV-2 ORF8, and caspase-6) are downloaded from the PDB database. PyMol software was used to remove ligands and non-protein molecules (such as water) in proteins. The 2D structures of core ingredients in the “drug-ingredient-target” network were downloaded from the TCMSP database. Finally, molecular docking was performed using AutoDock Vina, with SARS-CoV-2-related proteins as the receptors and core ingredients as the ligands (16). The molecular docking patterns were visualized *via* PyMOL.

### Statistical analysis

Graphpad Prism 9.0 was used for statistical analysis. Categorical data were described by the number of cases and constituent ratios, and comparisons between groups were performed by the chi-square test. Measurement data were expressed as mean ± standard deviation, and *t*-test was used for comparison between groups. Besides, a correlation analysis between negative PCR conversion days and leukocytes/lymphocytes/CRP, and hospitalization days and leukocytes/lymphocytes/CRP.

## Results

### Demographics data of COVID-19 patients from overseas

**Figure 1:**
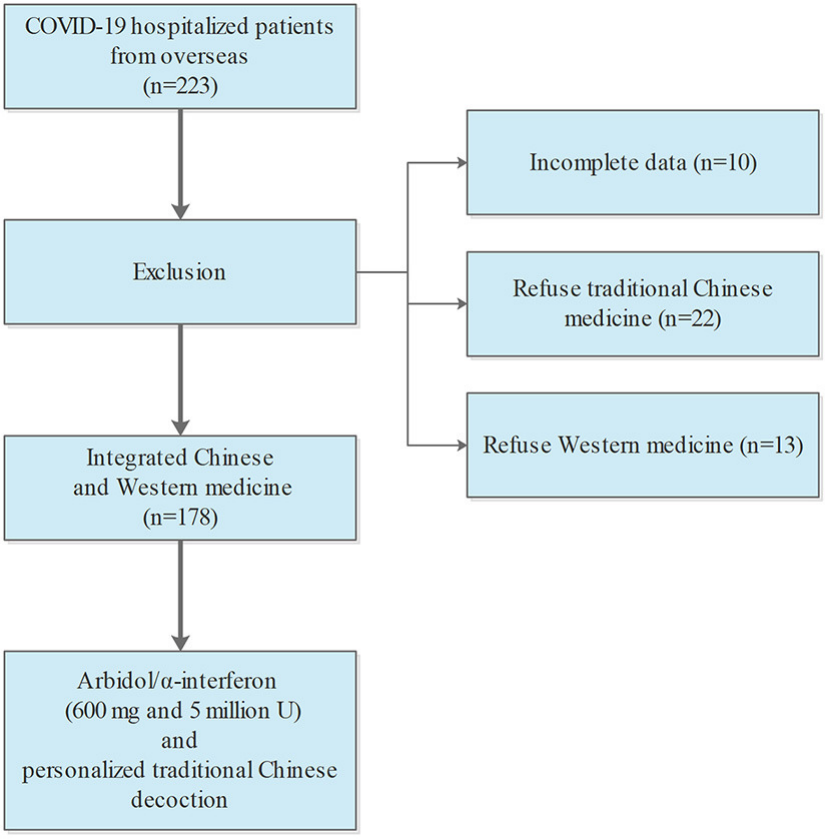
Flowchart showing the COVID-19 patients from overseas who obtained the therapy of integrated Chinese medicine and Western medicine.

Between April 1, 2021, and July 31, 223 adult patients with COVID-19 infection were admitted. Twenty-two patients gave up Chinese medicine treatment, 13 patients gave up Western medicine treatment. Ten patients with incomplete data were excluded from the study (Figure 1). Therefore, a total of 178 patients were included (median age 42.0 years; range 6–66 years; 60.1% female; Table 1). The BMI was 22.48 among the patients. Females had lower BMI (21.7 vs. 25.0, *p* < 0.001) compared to males. There were 63 patients who had comorbidities, such as respiratory disease (4.5%), diabetes (5.1%), and cardiovascular disease (14.6%).

**Table 1:** Demographic results of infected subjects from overseas by gender.

	**All patients (*n* = 178)**	**Male (*n* = 71)**	**Female (*n* = 107)**	***P*-value**
Age, years	42.00 (12.35)	34.00 (10.42)	49.00 (11.93)	0.000
BMI, kg/m^2^	22.48 (20.82)	25.00 (32.40)	21.70 (2.58)	0.000
**Comorbidities (%)**				
Any	63 (35.4)	29 (40.8)	34 (31.8)	0.215
Respiratory disease	8 (4.5)	4 (5.6)	4 (3.7)	0.819
Diabetes	9 (5.1)	5 (7.0)	4 (3.7)	0.525
Cardiovascular disease	26 (14.6)	11 (15.5)	15 (14.0)	0.785
Tumor	0 (0.0)	0 (0.0)	0 (0.0)	
**Country or Region (%)**				
Taiwan, China	83 (46.6)	4 (5.6)	79 (73.8)	0.000
Philippines	19 (10.7)	14 (19.7)	5 (4.7)	0.001
U.S.	15 (8.4)	10 (14.1)	5 (4.7)	0.027
Cambodia	13 (13.7)	11 (15.5)	2 (1.9)	0.001
U.K.	10 (5.6)	6 (8.5)	4 (3.7)	0.315
Ghana	8 (4.5)	8 (11.3)	0 (0.0)	0.001
Malaysia	6 (3.4)	5 (7.0)	1 (0.9)	0.074
Thailand	5 (14.6)	3 (4.2)	2 (1.9)	0.639
Netherlands	4 (2.8)	1 (1.4)	3 (2.8)	0.921
Singapore	3 (1.7)	1 (1.4)	2 (1.9)	1.000
Canada	2 (1.1)	0 (0.0)	2 (1.9)	0.518
Ukraine	2 (1.1)	2 (2.8)	0 (0.0)	0.158
Kazakhstan	2 (1.1)	2 (2.8)	0 (0.0)	0.158
Serbia	2 (1.1)	2 (2.8)	0 (0.0)	0.158
Romania	1 (0.6)	0 (0.0)	1 (1.9)	1.000
India	1 (0.6)	1 (1.4)	0 (0.0)	0.399
Ireland	1 (0.6)	0 (0.0)	1 (1.9)	1.000
Georgia	1 (0.6)	1 (1.4)	0 (0.0)	0.399

Data are expressed by mean (SD) or n (%). P-values were calculated by χ^2^-test.

### Clinical characteristics of COVID-19 patients from overseas

On admission, the most common symptoms in COVID-19 patients were sputum and cough, followed with insomnia, fever and stuffy nose (Table 2). A total of 40 infected subjects (22.5%) were asymptomatic and 138 subjects (77.5%) were symptomatic (Table 3). The symptomatic subjects included 11 mild (6.2%), 126 moderate (70.8%), and 1 severe (0.6%). According to the chest CT measurement, abnormal lung findings were observed in 145 subjects, including 57 lateral and 88 bilateral abnormal (Table 4). The most abnormal lung characteristics were ground-glass opacities (34.8%), patchy shadow (14.7), and infiltration (24.2%). There was no significant difference between male and female infected subjects.

**Table 2:** Clinical symptoms of symptomatic subjects from overseas by gender.

	**All patients (*n* = 138)**	**Male (*n* = 50)**	**Female (*n* = 88)**	***P-*value**
Cough	36 (26.1)	14 (28.0)	22 (25.0)	0.700
Sputum	38 (27.5)	16 (32.0)	22 (25.0)	0.376
Fever	13 (9.4)	7 (14.0)	6 (6.8)	0.278
Stuffy nose	10 (7.2)	4 (8.0)	6 (6.8)	1.000
Runny nose	8 (5.8)	2 (4.0)	6 (6.8)	0.763
Chest tightness	9 (6.5)	2 (4.0)	7 (8.0)	0.585
Insomnia	18 (13.0)	5 (10.0)	13 (14.8)	0.424
Fatigue	6 (4.3)	3 (6.0)	3 (3.4)	0.777

Data are expressed by n (%). P-values were calculated by χ^2^-test.

**Table 3:** Distribution of disease severity of infected subjects from overseas by gender.

	**All patients (*n* = 178)**	**Male (*n* = 71)**	**Female (*n* = 107)**	***P*-value**
Asymptomatic	40 (22.5)	21 (29.6)	19 (17.8)	0.064
Mild	11 (6.2)	4 (5.6)	7 (6.5)	1.000
Moderate	126 (70.8)	45 (63.4)	81 (75.7)	0.077
Severe	1 (0.6)	1 (1.4)	0 (0)	0.836

Data are expressed by n (%). P-values were calculated by χ^2^-test.

**Table 4:** Computed tomography (CT) and blood characteristics of infected subjects from overseas by gender.

	**All patients (*n* = 178)**	**Male (*n* = 71)**	**Female (*n* = 107)**	***P*-value**
**Computed tomography findings on admission (%)**			
Any abnormal lung findings	145 (81.5)	56 (78.9)	89 (83.2)	0.469
Lateral	57 (32.0)	23 (32.4)	34 (31.8)	0.931
Bilateral	88 (49.4)	33 (46.5)	55 (51.4)	0.520
Ground-glass opacities	62 (34.8)	22 (31.0)	40 (37.4)	0.380
Patchy shadow	31 (17.4)	10 (14.1)	21 (19.6)	0.340
Infiltration	43 (24.2)	17 (23.9)	26 (24.3)	0.957
**Blood measurements**				
Leukocytes, × 10^9^	6.14 (1.76)	6.38 (1.82)	5.98 (1.70)	0.138
Lymphocytes, × 10^9^	1.95 (0.73)	2.03 (0.87)	1.90 (0.63)	0.386
CRP, mg/L	4.11 (9.68)	6.54 (12.74)	2.59 (6.76)	0.000

Data are expressed by n (%) or mean (SD). P-values were calculated by χ^2^-test or t-test.

### The effectiveness of integrated Chinese and western medicine in COVID-19 patients from overseas

**Figure 2:**
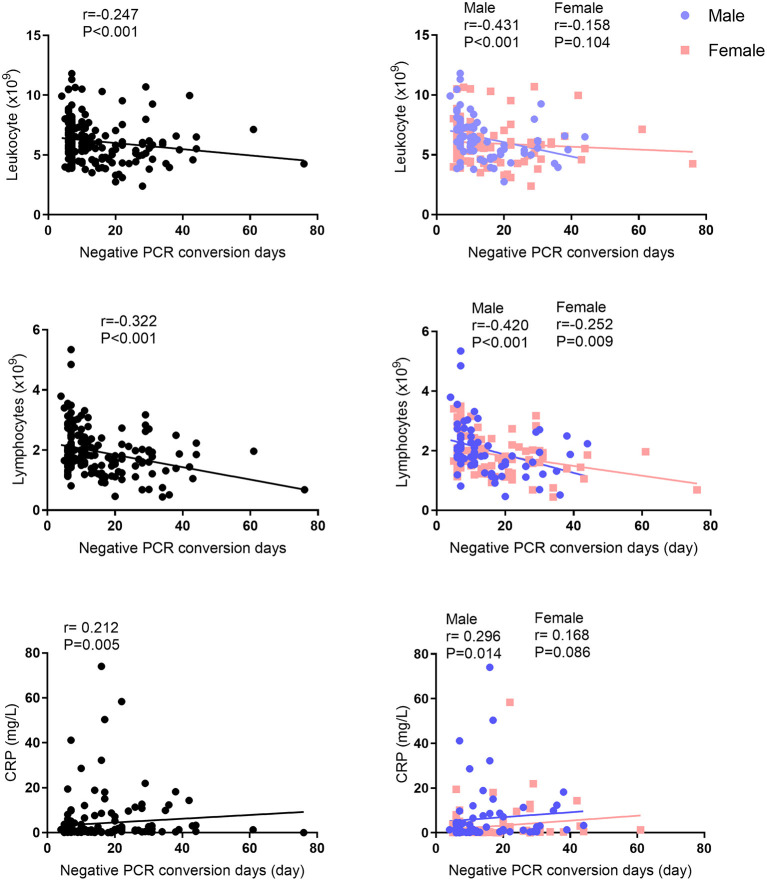
Correlation analysis between negative PCR conversion days and leukocytes, lymphocytes, or CRP levels.

**Figure 3:**
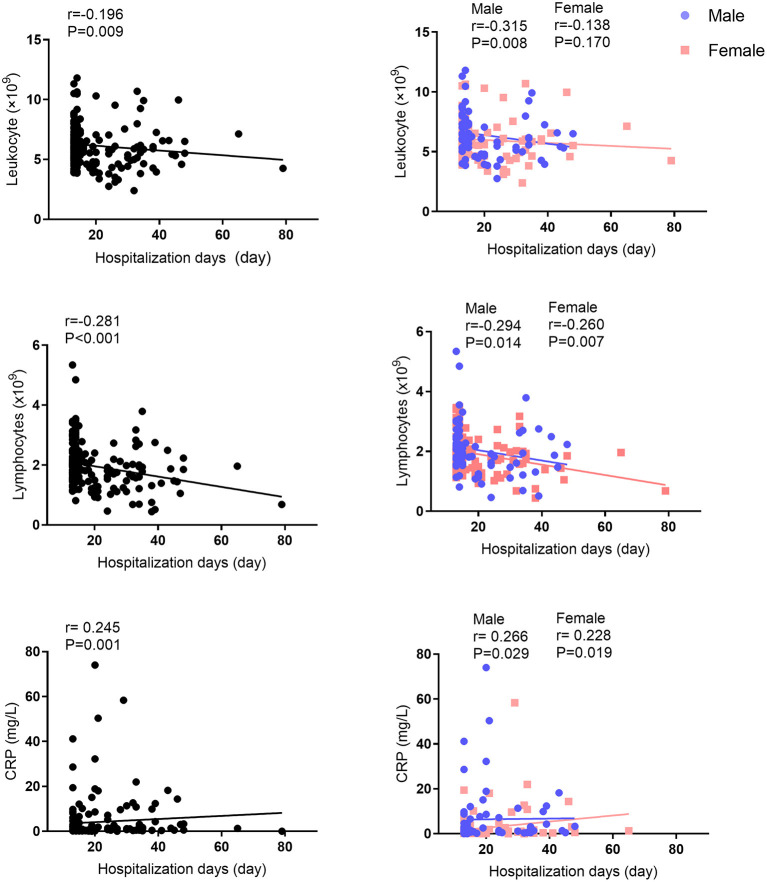
Correlation analysis between hospitalization days and leukocytes, lymphocytes, or CRP levels.

The median of nucleic acid conversion time was 7.0 days for asymptomatic subjects, 14.0 days for mild subjects, 10.0 days for moderate subjects, and 14.0 days for severe subjects (Table 5). In addition, the median length of hospitalization days was 7.0 days for asymptomatic subjects, 24.0 days for mild subjects, 15.0 days for moderate subjects, and 20.0 days for severe subjects. There was no difference between males and females. According to the correlation analysis, there are significant differences between negative PCR conversion days and leukocytes/lymphocytes/CRP (Figure 2). This significance of correlation was also observed in male. However, only the number of lymphocytes was correlated with negative PCR conversion days in female. On the other hand, there are significant differences between hospitalization days and leukocytes/lymphocytes/CRP (Figure 3). This significance of correlation was also observed in male. However, the number of leukocytes was not correlated with hospitalization days in female.

**Table 5:** Negative PCR conversion days and hospitalization days of infected subjects from overseas by gender.

	**All patients (*n* = 178)**	**Male (*n* = 71)**	**Female (*n* = 107)**	***P*-value**
**Negative PCR conversion days**				
All	10 (11.30)	11.0 (9.73)	10.0 (12.28)	0.285
Asymptomatic	7.0 (6.85)	10.0 (6.14)	7.0 (7.72)	0.346
Mild	17.0 (19.54)	16.5 (3.74)	28.0 (23.84)	0.354
Moderate	10.0 (11.05)	11.0 (11.01)	10.0 (11.01)	0.161
Severe	14.0	14.0		
**Hospitalization days**				
All	14.0 (10.78)	15.0 (9.84)	14.0 (11.41)	0.379
**Asymptomatic**	14.0 (6.53)	14.0 (6.46)	14.0 (6.70)	0.019
Mild	24.0 (18.17)	22.0 (2.63)	32.0 (22.02)	0.354
Moderate	15.0 (10.60)	15.0 (11.25)	15.0 (10.24)	0.602
Severe	20.0	20.0		

Data are expressed by median (SD). P-values were calculated by t-test.

### The frequency of Chinese medicine in COVID-19 patients from overseas

All patients were treated with personalized Chinese medicine. Therefore, there were 178 prescriptions for the treatment of COVID-19 patients from overseas. By screening and analyzing the prescriptions that induced less length of PCR positive days and hospitalization days than the median, we found that the top 4 frequently used Chinese medicine used in these prescriptions were *Scutellaria baicalensis* (Huangqin, 64 times), *Glycyrrhiza uralensis* (Gancao, 63 times)*, Bupleurum chinense* (Chaihu, 62 times), and *Pinellia ternata* (Banxia, 61 times). The frequency of the treatment with Chinese medicine in the top 10 is shown in Table 6.

**Table 6:** Frequency of Chinese medicine using in patients whose PCR positive length and hospitalization length less than median (top 10).

**Herbal medicine**	**All patients (*n* = 64)**	**Male (*n* = 22)**	**Female (*n* = 42)**	***P*-value**
*Scutellaria baicalensis*	64 (100)	22 (100)	42 (100)	
*Glycyrrhiza uralensis*	63 (98.4)	22 (100)	39 (92.9)	0.508
*Bupleurum chinense*	62 (96.9)	21 (95.5)	41 (97.6)	1.000
*Pinellia ternata*	61 (95.3)	22 (100)	39 (92.9)	0.508
*Dryopteris crassirhizoma*	58 (90.6)	21 (95.5)	37 (88.1)	0.612
*Atractylodes macrocephala*	44 (68.8)	16 (72.7)	28 (66.7)	0.619
*Poria cocos*	43 (67.2)	10 (45.5)	33 (78.6)	0.007
*Platycodon grandiflorum*	39 (60.9)	11 (50)	28 (66.7)	0.194
*Astragalus membranaceus*	36 (56.3)	9 (40.9)	27 (64.3)	0.073
*Citrus reticulata*	35 (54.7)	8 (36.4)	27 (64.3)	0.033

Data are expressed by n (%). P-values were calculated by χ^2^-test.

### Identification of active compounds and target genes of the clinical Chinese medicine in the treatment of COVID-19

**Figure 4:**
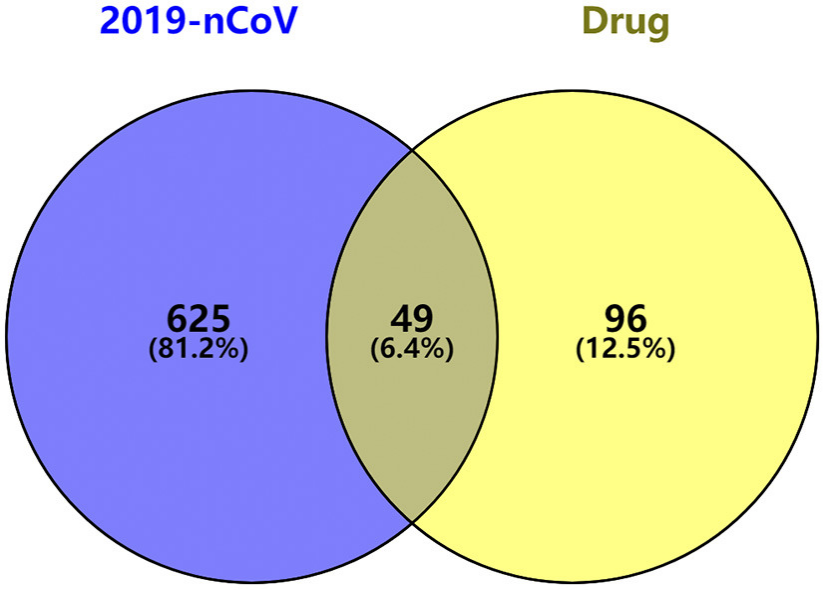
Venn analysis of top four drug target genes and COVID-19 related genes.

Based on the TCMSP and PharmMapper database threshold, active ingredients and targets of the top four Chinese medicines used in prescriptions were searched. Thirty-six ingredients of *Scutellaria baicalensis*, 92 ingredients of *Glycyrrhiza uralensis*, 17 ingredients of *Bupleurum chinense* and 13 ingredients of *Pinellia ternata* were obtained, and 145 genes were retained after removing duplicates from the targets. GeneCards and DisGeNet database were searched with “Novel Coronavirus Pneumonia” “viral pneumonia” “COVID-19” “2019-nCoV” to obtain the COVID-19 related genes, and 674 COVID-19 related genes were retained after removing duplicate items. The Venn diagram was drawn based on drug targets and COVID-19 related genes, and 49 anti-COVID-19 genes were obtained (Figure 4).

### Construction of protein-protein interaction (PPI) and drugs-ingredients-targets network

**Figure 5:**
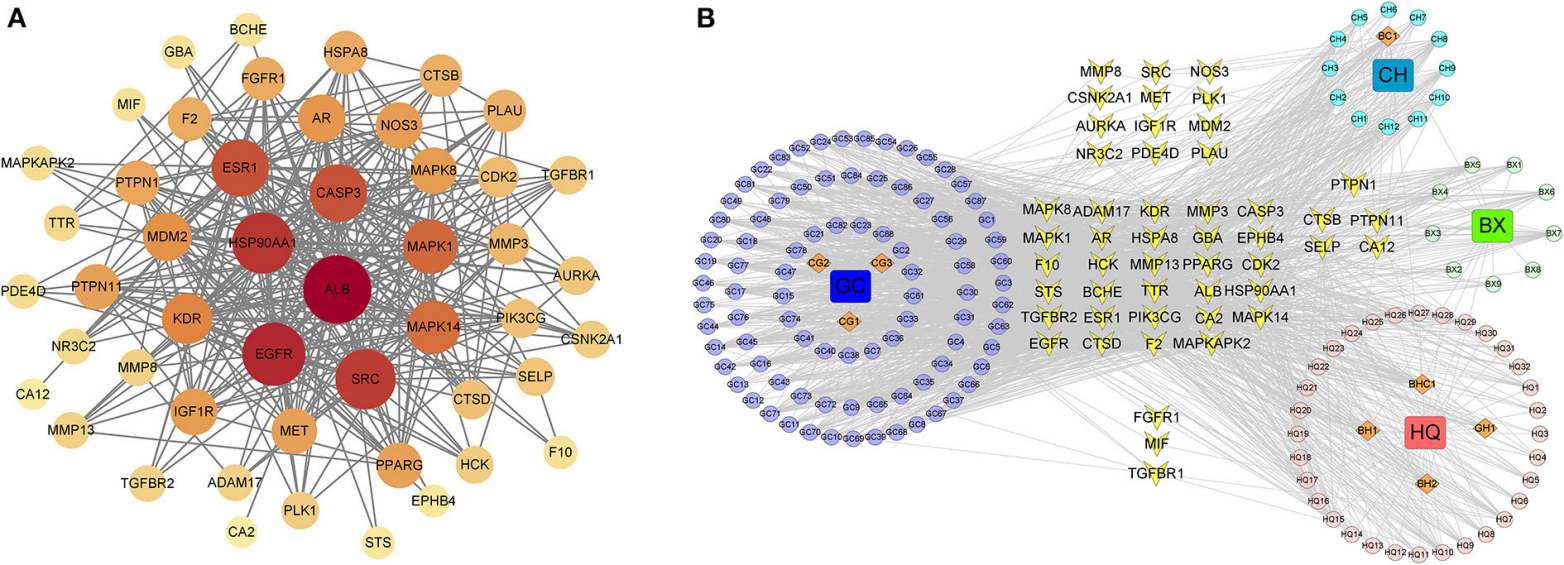
**(A)** Protein–protein network (PPI) of anti-COVID-19 genes. **(B)** Drugs-ingredients-targets network. The four squares represent drugs, BX (*Scutellaria baicalensis*), GC (*Glycyrrhiza uralensis*), CH (*Bupleurum chinense*), and BX (*Pinellia ternata*). The circles indicate the corresponding ingredients, the orange diamonds indicate the shared ingredient, BH(BX,HQ), BHC(BX,HQ,CH), BC(BX,CH), CG(CH,GC), GH(GC,HQ), and the yellow quadrilaterals represent targets.

The 49 anti-COVID-19 genes were analyzed by STRING to construct PPI network. It had 49 nodes and 301 edges (Figure 5A), and the average node degree was 12.3. The size and color of the targets were positively correlated with the degree, indicating that the bigger and darker targets were more closely connected. The targets with no correlation were hidden and plotted by Cytoscape, and the results of the topological analysis are shown (Table 7). According to the analysis of the results, the targets such as ALB, EGFR, HSP90AA1, and SRC interact with multiple targets and maybe the core targets. Four drugs, 158 ingredients and 49 targets were imported into Cytoscape to construct the Drugs-Ingredients-Targets network (Figure 5B). The network indicated that the Chinese medicine treatment has the characteristics of multi-ingredient and multi-target. Based on the results of a topological analysis, core ingredients (Baicalin, Sigmoidin-B, Stigmasterol, Troxerutin) can be screened out, which may be the key ingredients for the treatment of COVID-19 (Table 8).

**Table 7:** Information of 10 core genes after protein–protein network analysis.

**UniProt ID**	**Gene symbol**	**Protein name**	**Degree**
P02768	ALB	Albumin	38
P00533	EGFR	Epidermal growth factor receptor	33
P07900	HSP90AA1	Heat shock protein HSP 90-alpha	31
P12931	SRC	Proto-oncogene tyrosine-protein kinase Src	30
P03372	ESR1	Estrogen receptor	27
P42574	CASP3	Caspase-3	27
Q499G7	MAPK1	Mitogen-activated protein kinase	24
Q16539	MAPK14	Mitogen-activated protein kinase 14	23
P35968	KDR	Vascular endothelial growth factor receptor 2	20
Q00987	MDM2	E3 ubiquitin-protein ligase Mdm2	18

**Table 8:** Information of 10 core ingredients and target genes after drugs-ingredients-targets network analysis.

**Symbol**	**MOL-ID**	**Ingredient**	**Drug**	**Degree**
BC1	MOL002776	Baicalin	*Pinellia ternata, Bupleurum chinense*	32
GC59	MOL004935	Sigmoidin-B	*Glycyrrhiza uralensis*	27
BHC1	MOL000449	Stigmasterol	*Pinellia ternata, Scutellaria baicalensis, Bupleurum chinense*	26
CH8	MOL004648	Troxerutin	*Bupleurum chinense*	26
CH12	MOL013187	Cubebin	*Bupleurum chinense*	26
BX7	MOL006937	12,13-epoxy-9-hydroxynonadeca-7,10-dienoic acid	*Pinellia ternata*	25
CG2	MOL000354	Isorhamnetin	*Bupleurum chinense, Glycyrrhiza uralensis*	24
GC63	MOL004949	Isolicoflavonol	*Glycyrrhiza uralensis*	24
GC79	MOL005001	Gancaonin H	*Glycyrrhiza uralensis*	24
GC17	MOL004810	Glyasperin F	*Glycyrrhiza uralensis*	23

### Gene ontology (GO) and Kyoto encyclopedia of genes and genomes (KEGG) analysis

**Figure 6:**
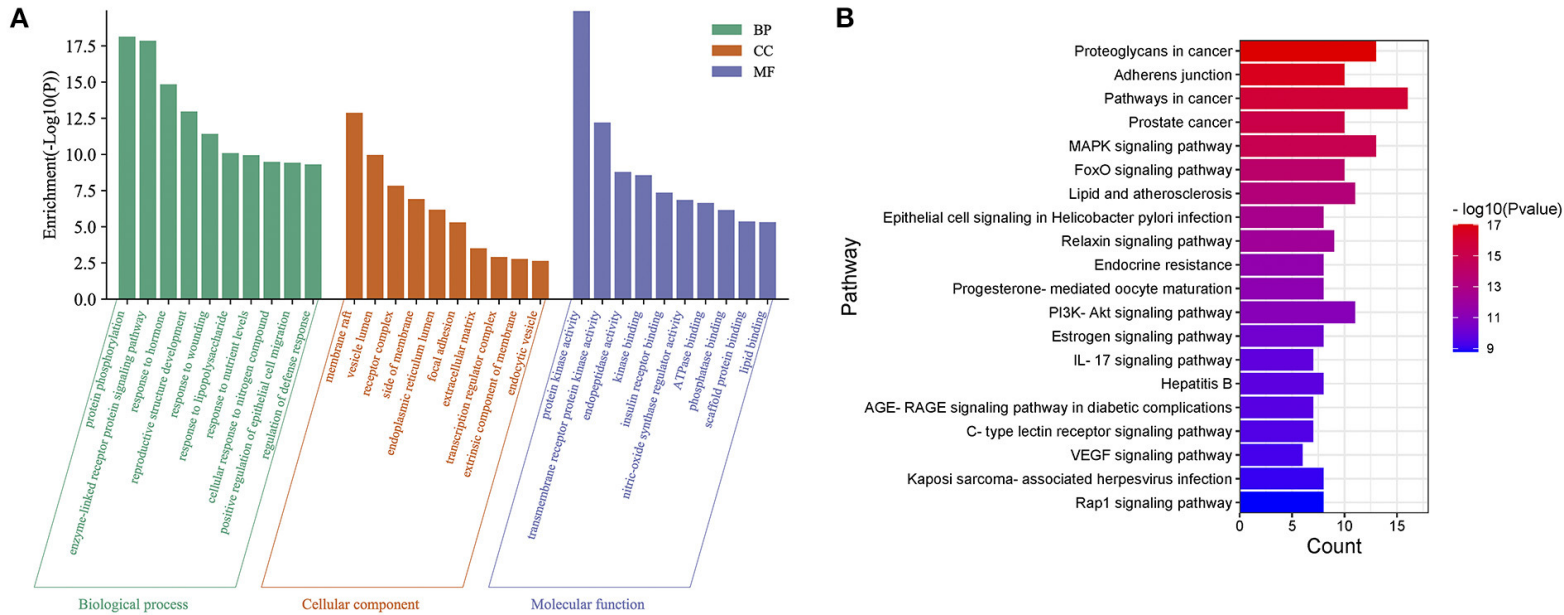
**(A)** GO enrichment analysis of potential target genes for COVID-19. **(B)** KEGG enrichment analysis of potential target genes for COVID-19.

The results of GO and KEGG enrichment analysis for 49 targets were obtained from the Metascape database. The top 10 terms in the three groups of BP, CC and MF were plotted in a bar chart (Figure 6A). The BP enrichment mainly included protein phosphorylation, enzyme-linked receptor protein signaling pathway, response to hormone, reproductive structure development, and response to lipopolysaccharide. The CC enrichment mainly included membrane raft, vesicle lumen, and receptor complex. The MF enrichment mainly included protein kinase activity, transmembrane receptor protein kinase activity, endopeptidase activity, and kinase binding. In addition, the top 20 entries of the KEGG enrichment analysis were plotted (Figure 6B). It mainly included Proteoglycans in cancer, Adherens junction, MAPK signaling pathway, FoxO signaling pathway, and Lipid and atherosclerosis.

### The binding affinity between active ingredients and potential targets

**Figure 7:**
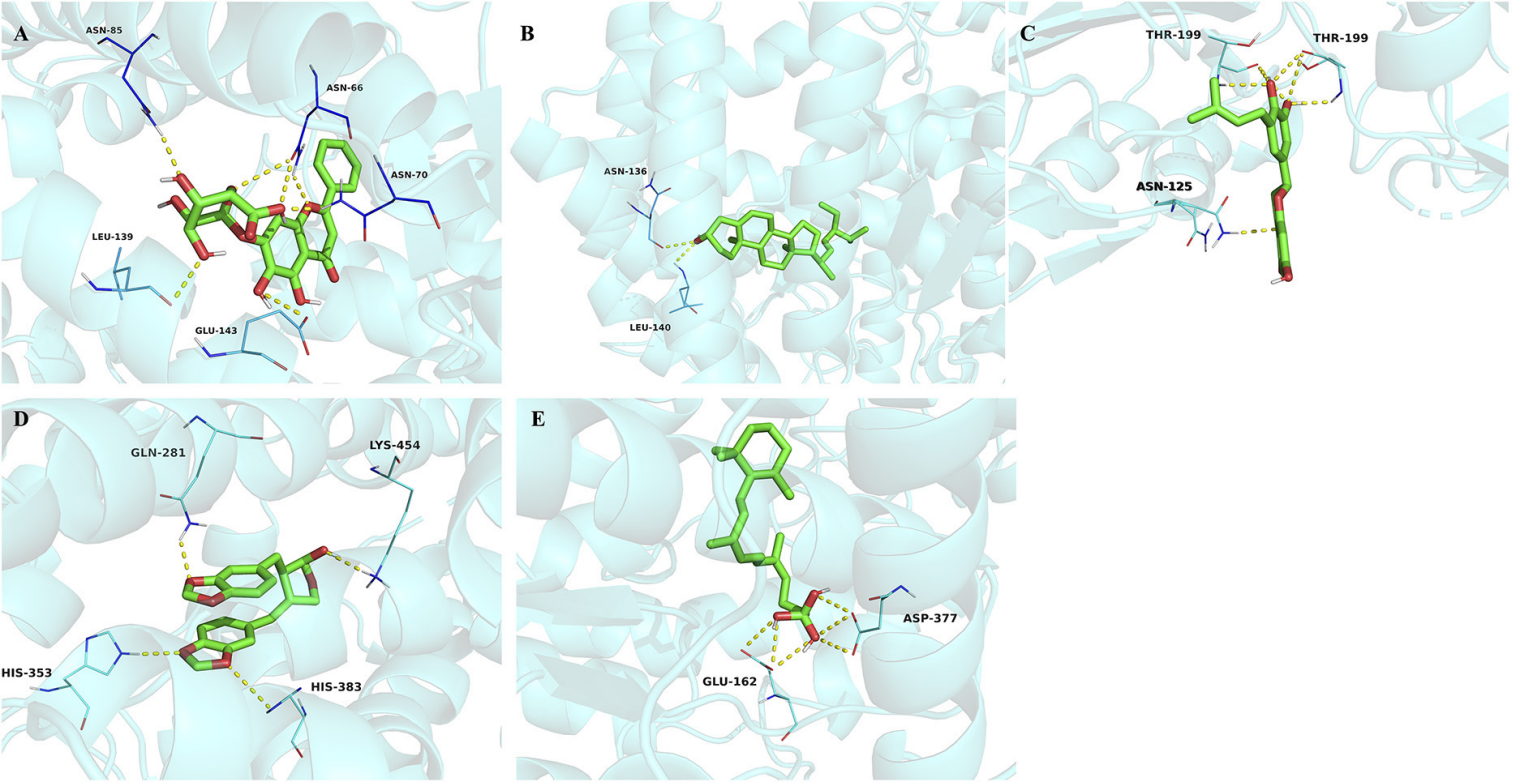
**(A)** Molecular docking of baicalin binding with ACE. **(B)** Molecular docking of stigmasterol binding with ACE. **(C)** Molecular docking of sigmoidin-B to caspase-6. **(D)** Molecular docking of cubebin binding with ACE. **(E)** Molecular docking of troxerutin binding with ACE. The yellow dot lines indicate the hydrogen bonds.

The top 5 active ingredients (Baicalin, Stigmasterol, Sigmoidin-B, Cubebin, Troxerutin) in the “drugs-ingredients-targets” network and SARS-CoV-2 related genes were selected, and the docking score was calculated (Table 9). In general, the lower docking score indicates that the ingredient plays a role in treating SARS-CoV-2. The results showed that the average docking score of each ingredient docked with the target protein was <-7.0 kcal/mol, indicating that the ingredient binds tightly to the target protein. We found that Baicalin and Stigmasterol are the same ingredients in medicines (Pinellia ternate, Bupleurum chinense), and the average docking score is higher. The components with the lower docking score were visualized with gene combinations with the PyMol software (Figure 7). These ingredients formed 2–7 hydrogen bonds with residues, implying that the interaction was stable.

**Table 9:** Docking score of effective ingredients to SARS-CoV-2 related genes.

**Ingredients**	**Baicalin**	**Stigmasterol**	**Sigmoidin-B**	**Cubebin**	**Troxerutin**
ACE	−9.3	−9.3	−9.4	−8.7	−8.1
ACE2	−6.9	−6.7	−7.6	−7.2	−7
SARS-CoV-2 3CL	−7.7	−7.0	−8.2	−6.8	−6.7
SARS-CoV-2 helicase-NSP13	−9.1	−8.1	−8.6	−7.4	−7.0
SARS-CoV-2 Spike	−7.8	−8.3	−8.3	−7.7	−7.3
SARS-CoV-2 NSP9	−6.5	−7.0	−7.2	−6.9	−6.6
SARS-CoV-2 Nucleocapsid Phosphoprotein	−7.4	−7.8	−8.0	−8.0	−6.6
SARS-CoV-2 ORF3a	−8.4	−7.4	−8.2	−7.8	−6.9
SARS-CoV-2 ORF7a	−8.1	−7.9	−8.1	−8.7	−7.3
SARS-CoV-2 ORF8	−7.1	−6.8	−7.1	−6.2	−6.0
Caspase-6	−9.0	−6.3	−9.6	−8.0	−8.0
Average	−7.9	−7.5	−8.2	−7.6	−7.0

## Discussion

People infected with SARS-CoV-2 usually have a dry cough and fever, accompanied by symptoms of general weakness (17). In some cases, SARS-CoV-2 causes the symptoms of diarrhea, nasal congestion, and runny nose (18). The disease progresses very rapidly after infection, and in about a week, breathing will be difficult, the body temperature will continue to rise, and there will be obvious and severe coughing, expectoration, and chest pain, headache, dizziness and other symptoms (19). In the present study, the main clinical manifestations of COVID-19 patients were sputum, cough, insomnia, fever, and stuffy nose, parallel to the recent studies (20, 21).

The COVID-19 patients collected in this study came from 18 countries or regions. Among these 178 patients, 14.6% had cardiovascular disease, 5.1% had diabetes, 4.5% had respiratory disease, and 2.3% had cerebrovascular disease. According to CT checks of the patients on admission, 32.0% had lateral abnormalities, 49.4% had bilateral abnormalities. There is no difference in symptoms between males and females. After treating with integrated Chinese medicine and Western medicine, the results showed that the median time of PCR conversion was 10 days, and the median of hospitalization time was 14 days. A previous study showed nasopharyngeal specimen negative conversion time, and the length of hospitalization of COVID-19 patients in the Arbidol group was 18.50 and 23.52 days, respectively (22). Another study showed that the mean duration of viral clearance was 23.42 days (23). Similarly, a recent retrospective cohort study indicated that the median of hospitalization time of Western medicine therapy (Antiviral plus interferon) was 21 days (24), which was longer than the integrated Chinese medicine and Western medicine in the present study. In Gabon, COVID-19 patients received an integrated therapy including oxygen, hydroxychloroquine, azithromycin, vitamin C, zinc tablet, and dexchlorpheniramine. The researchers found that the median duration of viral clearance was 14 days (25), while the median duration of viral clearance was 10 days in the present study. These data showed that integrated Chinese medicine and Western medicine are effective in treating COVID-19 patients.

Next, we summarize the Chinese medicine prescription, which makes the nucleic acid conversion time and hospitalization time less than the median. It was found that the top 10 Chinese medicine frequently used almost belonged to the category of heat-clearing and detoxifying, and their efficacy was mainly focused on clearing heat and detoxifying, drying dampness and resolving phlegm, lowering rebellion and stopping vomiting, primarily belonging to the lung, spleen and stomach meridians. It is also mentioned in the ancient Chinese Medicine Publications *Wenrelun* and *Wenbingtiaobian* that the lung is the first place to be attacked by the plague, and the lung symptoms of cough, phlegm, and dry throat are similar to the clinical manifestations of patients with COVID-19.

The top four Chinese medicines frequently used were selected for the bioinformatic research. It consists of *Scutellaria baicalensis, Glycyrrhiza uralensis, Bupleurum chinense*, and *Pinellia ternate*. Coincidentally, the composition of these four medicines is similar to the well-known classical TCM formula *Xiaochaihutang*. *Xiaochaihutang* can regulate the qi activity, harmonize and lease the less yang. Clinically, the use of *Xiaochaihutang* for COVID-19 patients with high fever often achieves satisfactory antipyretic effects. Network pharmacological studies have also confirmed that *Xiaochaihutang* can treat COVID-19 by inhibiting the viral infection of host cells and self-replication, inhibiting cytokine release syndrome, and improving hypoxemia (26).

Then, we collected the relevant ingredients and targets of four Chinese medicine through the network pharmacology study. We also performed PPI analysis, network construction, topology analysis, and pathway enrichment analysis on the targets. The study identified five core components, Baicalin, Stigmasterol, Sigmoidin-B, Cubebin, and Troxerutin, which mainly belong to flavonoids. According to the results of PPI, the core targets contain ALB, EGFR, HSP90AA1, and SRC. Next, GO functional analysis of anti-COVID-19 genes showed enrichment in protein kinase activity, endopeptidase activity, and nitric-oxide synthase regulator activity. KEGG pathway analysis showed major involvement in Proteoglycans in cancer, Adherens junction, IL 17 signaling pathway, MAPK signaling pathway, and FoxO signaling pathway. Finally, molecular docking was applied to predict the five core ingredients with SARS-CoV-2-related targets. These proteins play important roles in viral replication, transcription, cell cycle blocking, and mediating immune regulation in SARS-CoV-2 virus-host infection and were therefore selected for molecular docking of COVID-19 (27). The results revealed that ACE and caspase-6 showed low binding energy to the five core ingredients, indicating that they could be used as targets for molecular therapy. Among them, stigmasterol and baicalin in *Pinellia ternata, Bupleurum chinense*, and *Scutellaria baicalensis* had the lowest binding energy to ACE, and sigmoidin-B in *Glycyrrhiza uralensis* had the lowest binding energy to caspase-6.

Cytokine storm is a systemic inflammatory response, which can be caused by multiple factors, such as pathogens and excessive activation of immune cells, manifested by a sharp increase in the levels of various inflammatory factors (28). It has been found that COVID-19 patients have varying degrees of cytokine storm, which causes an inflammatory immune response and is a key factor in poor patient survival (29). We found that studies of the core components and targets showed anti-inflammatory activity in inflammation and immune regulation. Baicalin can be widely used in inflammatory responses caused by viral infections, such as inhibition of pro-inflammatory cytokines (TNF-α, IL-6) in influenza A (30), inhibition of SARS-CoV-2 RNA-dependent RNA polymerase (RdRp), and angiotensin-converting enzyme 2 (ACE2) activity to prevent SARS-CoV-2 from entering the host cells (31, 32). Stigmasterol exerts potential anti-inflammatory effects by inhibiting the IL-1beta-induced NF-kappaB pathway, suppressing pro-inflammatory cytokine expression, and prostaglandin E2 [PGE(2)] production (33). Troxerutin prevents inflammatory responses by reducing the levels of inflammatory cytokines such as IL-10, TNF-α, and IL-6. In addition, it inhibits the activation of procaspase-9 and procaspase-3, reduces the production of ROS, and enhances antioxidant activity (34). Pro-inflammatory cytokines such as TNF-α and IL-6 inhibit the production of ALB. The meta-analysis also showed that elevated CRP levels and decreased ALB levels were the most common laboratory findings in patients with COVID-19 (35). Experiments show that ALB causes the upregulation of ACE and the downregulation of ACE2 by interrupting the balance of ACE and ACE2 (36). ACE/ACE2 are also associated with COVID-19 infection. During infection control, HSP90AA1 induces autophagy by interacting with the AKT-MTOR pathway after recognizing the virus (37). EGFR plays a role in SARS-CoV-2 virus replication and affects the host immune response. In addition, excessive activation of EGFR plays a role in the development of pulmonary fibrosis (38, 39). It was found that the severity of EGFR involvement and pulmonary fibrosis in patients who died from COVID-19 pneumonia correlated with CRP levels (40). The MAPK signaling pathway and IL 17 signaling pathway play an essential role in the inflammatory response by secreting pro-inflammatory cytokines such as TNF-α, IL-1, and IL-6, exacerbating the inflammatory response (41). PI3K-Akt and FoxO signaling pathways participate in cell proliferation, differentiation, oxidative stress, and apoptosis. It can inhibit the autophagy of lung fibroblasts and prevent the formation of pulmonary fibrosis in preventing and treating pneumonia (42, 43). It has been shown that caspase-6 cleaves the coronavirus nucleocapsid (N) proteins and is an important host factor capable of efficiently replicating coronaviruses. Lung lesions and weight loss in SARS-CoV-2 infected mice were substantially attenuated by inhibition of caspase-6 (44). After the ACE2 transfection of host cells, the replicative capacity of SARS-CoV increased (45). SARS-CoV-2 was able to downregulate ACE2 expression, elevate pro-inflammatory cytokine expression, and induce a Cytokine storm (46). The SARS-CoV-2 virus binds to ACE2 *via* the Spike protein, which has a stronger viral attachment, and the Spike protein facilitates the entry of SARS-CoV-2 virus into the human body (47). SARS-CoV-2 3CL protease manages viral maturation and replication, and by inhibiting its expression, it is a crucial drug target for COVID-19 therapy (48). The primary function of the Nucleocapsid protein is not only to replicate the virus but also to assemble the RNA of the viral genome into ribonucleoprotein complexes (49). ORF3a, ORF7a, and ORF8 are accessory proteins of CoVs that exhibit high variability. The accessory proteins do not play a role in viral replication but have an important role in host immune evasion (50, 51). The innate interferon (IFN) response constitutes one of the host's first lines of defense against viral infection. ORF7a inhibits IFN-I signaling by suppressing STAT1 phosphorylation (52).

However, it should be noted that there are several limitations to this study. First, this is a single-center retrospective non-randomized design. Selection bias cannot be avoided. In this respect, a double-blinded, prospective, randomized controlled clinical trial is required to fully elucidate the therapeutical effect of Chinse medicine. Second, the study did not follow up with patients and could not observe the long-term effects of Chinese medicine on COVID-19. Third, in terms of the underlying mechanism, this study focuses on network pharmacology and bioinformatics analysis. Therefore, more molecular experiments are needed to verify the efficacy of putative active ingredients against COVID-19 *via* targeting the potential targets.

In the context of the continuous mutation of the SARS-CoV-2 and the rising number of infected patients, we believe that Chinese medicine is an effective therapy for treating COVID-19. Our study, for the first time, systematically analyzes the efficacy of Chinese medicine in treating COVID-19 patients from overseas, and discusses the putative active ingredients and the potential targets of Chinese medicine. Therefore, the present study provides a clinical reference and theoretical basis for using Chinese medicine to treat COVID-19 in countries around the world.

## Data availability statement

The original contributions presented in the study are included in the article/supplementary material, further inquiries can be directed to the corresponding author/s.

## Ethics statement

The study was approved by the Ethics Review Committee of the First Affiliated Hospital of Xiamen University (No. 2021-045). Written informed consent for participation was not required for this study in accordance with the national legislation and the institutional requirements.

## Author contributions

X-QC conceived the project, designed the study, analyzed the data, and wrote the manuscript. N-FL, C-YL, F-PZ, X-YY, B-HL, and Q-MC collected the data. X-ZH, N-JZ, and J-YY analyzed the case. M-MZ analyzed the data. L-TY and Y-XH analyzed the data and wrote the manuscript. All authors read and approved the final manuscript.

## Funding

This work was supported by the Key Funding from Fujian Natural Science Foundation (2022J01420640) and Xiamen Science and Technology Bureau (3502Z2021YJ08).

## Conflict of interest

The authors declare that the research was conducted in the absence of any commercial or financial relationships that could be construed as a potential conflict of interest.

## Publisher's note

All claims expressed in this article are solely those of the authors and do not necessarily represent those of their affiliated organizations, or those of the publisher, the editors and the reviewers. Any product that may be evaluated in this article, or claim that may be made by its manufacturer, is not guaranteed or endorsed by the publisher.
